# Epigallocatechin-3-gallate remodels apolipoprotein A-I amyloid fibrils into soluble oligomers in the presence of heparin

**DOI:** 10.1074/jbc.RA118.002038

**Published:** 2018-05-31

**Authors:** David Townsend, Eleri Hughes, Geoffrey Akien, Katie L. Stewart, Sheena E. Radford, David Rochester, David A. Middleton

**Affiliations:** From the ‡ Department of Chemistry, Lancaster University, Lancaster LA1 4YB and; the § Astbury Centre for Structural Molecular Biology, School of Molecular and Cellular Biology, Faculty of Biological Sciences, University of Leeds, Leeds LS2 9JT, United Kingdom

**Keywords:** atherosclerosis, apolipoprotein, solid-state NMR, circular dichroism (CD), electron microscopy (EM), polyphenol, glycosaminoglycan, amyloid

## Abstract

Amyloid deposits of WT apolipoprotein A-I (apoA-I), the main protein component of high-density lipoprotein, accumulate in atherosclerotic plaques where they may contribute to coronary artery disease by increasing plaque burden and instability. Using CD analysis, solid-state NMR spectroscopy, and transmission EM, we report here a surprising cooperative effect of heparin and the green tea polyphenol (−)-epigallocatechin-3-gallate (EGCG), a known inhibitor and modulator of amyloid formation, on apoA-I fibrils. We found that heparin, a proxy for glycosaminoglycan (GAG) polysaccharides that co-localize ubiquitously with amyloid *in vivo*, accelerates the rate of apoA-I formation from monomeric protein and associates with insoluble fibrils. Mature, insoluble apoA-I fibrils bound EGCG (*K_D_* = 30 ± 3 μm; *B*_max_ = 40 ± 3 μm), but EGCG did not alter the kinetics of apoA-I amyloid assembly from monomer in the presence or absence of heparin. EGCG selectively increased the mobility of specific backbone and side-chain sites of apoA-I fibrils formed in the absence of heparin, but the fibrils largely retained their original morphology and remained insoluble. By contrast, fibrils formed in the presence of heparin were mobilized extensively by the addition of equimolar EGCG, and the fibrils were remodeled into soluble 20-nm-diameter oligomers with a largely α-helical structure that were nontoxic to human umbilical artery endothelial cells. These results argue for a protective effect of EGCG on apoA-I amyloid associated with atherosclerosis and suggest that EGCG-induced remodeling of amyloid may be tightly regulated by GAGs and other amyloid co-factors *in vivo*, depending on EGCG bioavailability.

## Introduction

Amyloidosis is a group of disorders characterized pathologically by the extracellular accumulation of insoluble protein fibrils with a cross-β structural motif. Systemic amyloidosis affects several organs and tissues, whereas localized amyloid is confined to a single organ. In each case the clinical manifestations depend on the precursor protein (1). Localized amyloid deposits of the Aβ^5^ peptide in Alzheimer's brains have been characterized extensively, although the relationship between misfolded Aβ and disease remains unresolved. The pathological consequences of systemic amyloidosis are less ambiguous and often involve irreparable damage to major organs at the end-stage of disease. Systemic amyloid derived from apolipoprotein A-I (apoA-I), the major protein component of the high-density lipoprotein (HDL) complex that transports cholesterol to the liver, was identified in the 1990s as a hereditary condition related to several mutant forms of the protein that are susceptible to protease digestion, misfolding, and aggregation (2, 3). More recent evidence has shown that fibrils of WT apoA-I also accumulate spontaneously in the plaques of atherosclerosis and may contribute to the condition (4–7). Lipid-deprived apoA-I can undergo an alternative folding pathway in which the protein self-assembles into amyloid (8, 9), resulting in loss of its atheroprotective properties and the accumulation of potentially damaging amyloid plaques in vital organs and vasculature. The high incidence of fibrillar apoA-I associated with atherosclerotic lesions suggests that amyloid deposition may decrease plaque stability and contribute to the progression of atherosclerosis (6, 7, 10, 11).

Native, functional apoA-I has a predominantly α-helical structure, but the fibrillar aggregates exhibit a combination of α-helical and β-sheet conformations as revealed by CD and solid-state NMR spectroscopy (12). ApoA-I aggregation *in vitro* is accelerated under acidic conditions (11) and by myeloperoxidase-catalyzed oxidation (13) associated with inflammatory diseases such as atherosclerosis. Whether the enhanced aggregation kinetics at low pH is pathologically significant is not clear, although a small but significant pH reduction (to 7.15) has been observed *ex vivo* in calcified areas of lesions (14). In addition, heparin, a member of the glycosaminoglycan (GAG) polysaccharides that co-localize ubiquitously with amyloid *in vivo* (15, 16), accelerates the formation of ordered apoA-I fibrils in a concentration-dependent manner (11, 12). Generally, heparin is known to increase the rate of amyloid fibril formation (17, 18), stabilize fibrils (19, 20), and reduce amyloid toxicity (21, 22). The GAG chains of proteoglycans in the arterial intima associate with apoA-I in the advanced stages of atherosclerosis (23), and the accumulation of high local apoA-I concentrations may contribute to the formation and retention of amyloid deposits. The native structure of apoA-I is stable for at least 24 h above pH 7 (12), but at pH 4–5 in the presence of heparin aggregation is virtually instantaneous.

Polyphenols from green tea, including (−)-epigallocatechin-3-gallate (EGCG), are flavonoids that are considered to have beneficial protective effects on cardiovascular health and against atherosclerosis, resulting from their anti-oxidant and anti-inflammatory properties (24, 25). EGCG has also been shown to modulate the aggregation kinetics of several amyloidogenic proteins, including Aβ, α-synuclein, and amylin, and directs the assembly pathway toward the formation of large, off-pathway, and nontoxic oligomers (26–28). EGCG remodels insoluble amyloid fibrils into amorphous aggregates with reduced toxicity to mammalian cells (29). Oxidized and unoxidized EGCG molecules bind to amyloid fibrils through engagement of hydrophobic sites (30) and polar contacts (31, 32), but autooxidation of EGCG appears to invoke covalent cross-linking with the fibrils that stabilize the remodeled aggregates (33). These properties of EGCG offer potential therapeutic benefits, and clinical trials of EGCG for the treatment of early-stage Alzheimer's and antibody light chain amyloidosis have been completed or are currently active. Low bioavailability and intestinal and hepatic metabolism are considered potential difficulties of medical utilization of the unmodified natural product (34, 35). Recently, it was shown that EGCG disaggregated fibrils of the G26R (Iowa) mutant of apoA-I and also inhibited fibril growth, as assessed by the amyloid-reactive dye thioflavin T (ThT) (36). The effects were also replicated on the N-terminal 1–83-amino acid peptide fragment of both the Iowa mutant and WT protein. A detailed molecular analysis of EGCG with WT apoA-I associated with atherosclerosis, however, has not been reported.

Here, we show that EGCG interacts with fibrils of WT, full-length apoA-I preferentially over other green tea components without modulating apoA-I fibril growth kinetics. Circular dichroism (CD), solid-state NMR spectroscopy (ssNMR), and transmission EM (TEM) reveal that EGCG remodels apoA-I fibrils into soluble nontoxic oligomers when fibrils are formed in the presence of heparin but not when fibrils are formed in the absence of heparin. This surprising synergistic effect of EGCG and heparin may offer a means of influencing the deposition of apoA-I amyloid associated with atherosclerosis and possibly other amyloids known to associate with GAGs *in vivo*.

## Results

### Heparin associates with apoA-I fibrils

**Figure 1.**
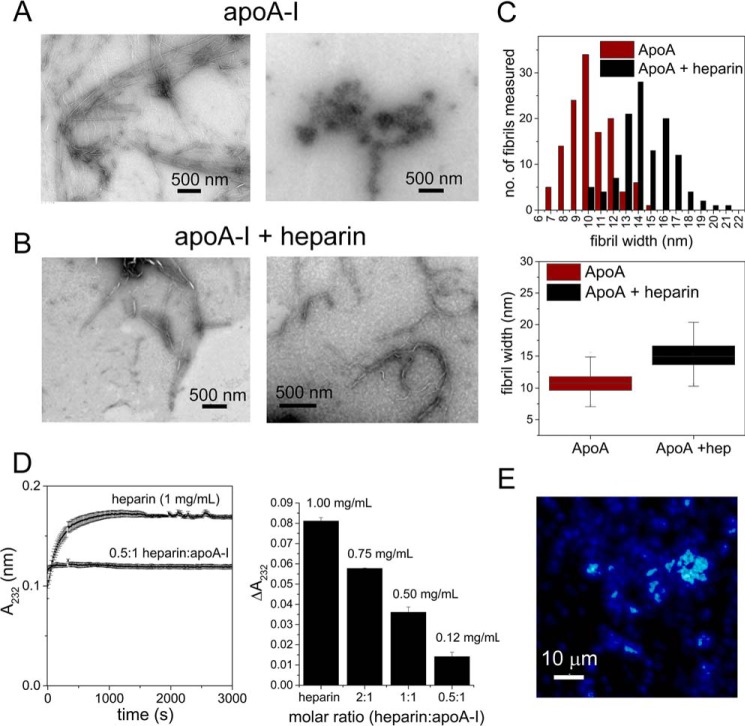
**ApoA-I fibril formation in the absence or presence of heparin.** *A,* negative stain TEM images of apoA-I aggregates formed at pH 4 in the absence of heparin. *B*, TEM images of apoA-I fibrils formed in the presence of 14–15 kDa of heparin (2-fold m excess). *C,* distribution and means of fibril widths measured from TEM images of apoA-I ± heparin. *D,* determination of heparin association with apoA-I fibrils. *Left*, time course of uronic acid generation resulting from heparin cleavage by heparinase I. *Right*, calculation of heparin remaining in solution following sedimentation with the protein at different molar ratios. Δ*A*_232_ was measured as the end point *A*_232_ value minus the initial *A*_232_. All initial solutions contained 1 mg/ml heparin (∼72 μm) before the addition of apoA-I and sedimentation of the aggregates formed. The concentrations shown represent the amount of heparin remaining in solution after removal of the insoluble material. *E,* fluorescence lifetime image (obtained on a Picoquant MicroTime 200 instrument operating at an excitation wavelength of 375 nm) of apoA-I fibrils formed with a 2-fold molar excess of heparin doped with 1% w/w of a heparin fluorescein conjugate (ThermoFisher Scientific). Fibrils were washed with aqueous buffer before sedimentation and dispersion onto glass coverslips. The *lower image* shows the total mean fluorescence lifetime corresponding to a best fitting biexponential curve of time constants 1.5 and 4.0 ns.

WT apoA-I is known to undergo ThT-responsive aggregation in solution at pH 4 that is accelerated by the addition of heparin (11, 12). TEM indicates that incubation of apoA-I alone at pH 4 for 3 days results in the deposition of fibrils (Fig. 1*A*, *left panel*) and distinct deposits of more amorphous material (Fig. 1*A*, *right panel*), whereas incubation of apoA-I with a 2-fold molar excess of heparin (assuming a mean mass of 14.5 kDa) results in only fibrils. The fibrils in the absence of heparin are 7–15 nm in diameter, and fibrils in the presence of heparin are distributed from 10 to 21 nm in diameter, although the mean widths are not significantly different (Fig. 1*C*). Heparin may therefore either influence the aggregation process and/or may co-localize with the fibrils, as found for other amyloid-forming proteins (37–41). None of the TEM images showed any evidence of nanoscale structures other than fibrils or amorphous aggregates, even after shorter incubation times.

Evidence for heparin binding to the fibrils was obtained using a heparinase I assay (42), in which the concentration of heparin remaining in solution was determined after incubation with monomeric apoA-I and removal of the fibrils formed after 3 days by sedimentation. From an initial concentration of 1 mg/ml, heparin is progressively removed from solution by increasing amounts of protein (Fig. 1*D*). Furthermore, apoA-I fibrils formed in the presence of heparin doped with a heparin–fluorescein conjugate showed strong fluorescence enhancement over the background protein fluorescence (Fig. 1*E*), consistent with co-localization of the fluorophore and fibrils. The results thus show that heparin binds to apoA-I during or after its aggregation into amyloid and precipitates concomitantly with the insoluble fibrils.

### Selective binding of green tea polyphenols to apoA-I fibrils

**Figure 2.**
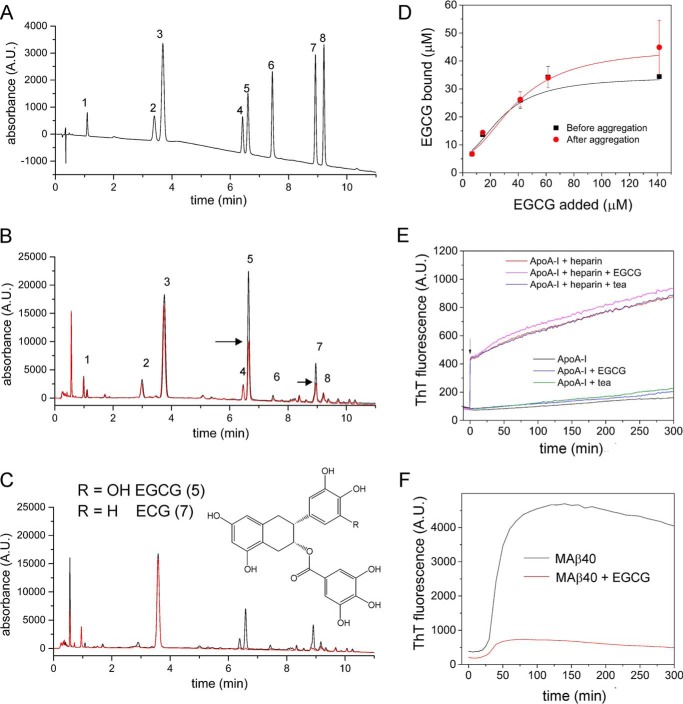
**Green tea catechin interactions with apoA-I fibrils.** *A,* reverse-phase HPLC analysis of a standard aqueous solution of eight green tea catechins: *1,* gallocatechin; *2,* caffeine; *3,* catechin; *4,* epicatechin; *5,* epigallocatechin-3-gallate (EGCG); *6,* gallocatechin-3-gallate; *7*, epicatechin-3-gallate; and *8*, catechin-3-gallate. *B,* HPLCs of microwave-extracted green tea solution (*black*) and the solution after the addition and removal by sedimentation of apoA-I fibrils (*red*). *Arrows* highlight the peak height reductions for compounds 5 and 7. *C,* chromatograms of green tea solution (*black*) and the solution after the addition and removal by sedimentation of MAβ40 fibrils (*red*). The *inset* shows the chemical structures of EGCG and ECG. *D,* binding of EGCG to apoA-I. EGCG was added to protein monomer (36 μm) before incubation (*black*) or to the preformed fibrils (1 mg/ml monomer equivalent; *red*) as described under “Materials and methods.” *Solid lines* represent the best-fitting Hill plots, yielding the values of *K_d_* and *B*_max_ as given in the text. *E,* ThT fluorescence for apoA-I incubated in the presence or absence of heparin (2-fold molar excess) and with the addition of EGCG or green tea extract, prior to acidification. The *arrow* denotes the addition of heparin. *F,* ThT fluorescence for Aβ40 fibrils incubated with EGCG. All binding curves and ThT plots represent the mean of three replicate samples.

To investigate whether apoA-I fibrils bind EGCG and other polyphenols from green tea, an aqueous green tea solution was prepared by microwave extraction of the dried leaves. A HPLC method was developed to quantify green tea polyphenol and caffeine binding to pre-formed fibrils. Insoluble fibrils prepared in the absence of heparin at pH 4 (1 mg/ml monomeric apoA-I) were suspended in an aqueous solution of green tea (1 ml) and incubated for 12 h with gentle agitation before centrifugation and retention of the supernatant for analysis. Control samples of the tea extract solution alone were treated in the same way. Reverse-phase HPLC of the control solution resolves several major components that were identified by MS (12) and quantified with reference to a standard mixture of eight green tea catechins of known concentration (Fig. 2 and Table 1). Comparison of the peaks for the fibril-treated and control solutions reveals that specific components are removed from solution by binding to the fibrils. The major component, EGCG (*peak 5*), reduces to <50% of its initial concentration after sedimentation of the apoA-I fibrils (Fig. 2*B*), and epicatechin-3-gallate (ECG) (peak 7) also shows appreciable binding. The 3-gallate moiety therefore appears to enhance the affinity of the polyphenols to associate with apoA-I fibrils. EGCG and ECG are also removed from the green tea extract when incubated with fibrils of the 40-amino acid β-amyloid peptide (Aβ40) (Fig. 2*C*), confirming the affinity of these polyphenols for amyloid fibrils derived from different proteins. Titration of apoA-I fibrils (36 μm monomer equivalent concentration) with a solution of pure EGCG yields an apparent dissociation constant (*K_d_*) and saturation binding concentration (*B*_max_) of 40 ± 4 and 45 ± 5 μm, respectively (Fig. 2*D*). Similar values (*K_d_* = 30 ± 3 μm; *B*_max_ = 35 ± 3 μm) were obtained when EGCG was added to the monomeric protein and incubated for 48 h before removing the insoluble protein by centrifugation. In both cases the saturation binding concentration of EGCG is approximately equimolar.

**Table 1:** **ApoA-I fibril binding of microwave-extracted green tea catechins as quantified from the HPLC peak intensities** Fibrils formed from 36 μm apoA-I were centrifuged and resuspended in 200 ml of green tea extract solution before further centrifugation and HPLC analysis of the supernatant.

Compound	Concentration		
	Initial*^a^*	Bound*^b^*	Bound
	μ*g/ml*	μ*g/ml*	%
1. Gallocatechin	337.1	7.3	2.2
2. Catechin	506.4	46.0	9.1
3. Caffeine	302.9	62.6	20.7
4. Epicatechin	154.9	0	0
5. Epigallocatechin-3-gallate	958.7	533.2	55.6
6. Gallocatechin-3-gallate*^c^*	35.2	24.8	70.6
7. Epicatechin-3-gallate	200.3	98.0	48.9
8. Catechin-3-gallate*^c^*	52.7	9.7	18.5

*^a^* Initial concentrations before addition of fibrils were estimated from a peak-by-peak comparison of the HPLCs of the microwave-extracted solution and the standard green tea solution.

*^b^* Bound concentrations are given as the initial concentrations minus the supernatant concentrations after addition and sedimentation of the fibrils.

*^c^* Approximate values only are shown, as concentrations were too low to measure accurately.

### EGCG does not alter the aggregation kinetics of apoA-I

We next used ThT fluorescence to determine whether EGCG influences the kinetics of aggregation of apoA-I under conditions previously reported to favor apoA-I assembly into amyloid (11, 12). At pH 4, incubation of apoA-I alone results in a slow enhancement of ThT fluorescence (Fig. 2*E*). The addition of heparin, however, results in a rapid enhancement of ThT fluorescence, followed by a further, more gradual increase (Fig. 2*E*) (in the absence of protein heparin does not affect ThT fluorescence (data not shown)). Previous work has shown that the rapid fluorescence enhancement induced by heparin reflects an increase in insoluble, partially fibrillar protein (12). A repeat of the measurements in the presence of EGCG (2-fold molar excess over the protein) or green tea extract (containing an estimated 2-fold molar excess of EGCG with respect to the protein concentration) showed that EGCG and the leaf extract do not affect the aggregation kinetics of apoA-I, regardless of whether heparin is present. This observation contrasts with the reported inhibitory effect of EGCG on a peptide fragment of the apoA_Iowa_ variant (36) and on the β-amyloid peptide (29, 43). Equimolar EGCG is sufficient to cause a marked reduction in ThT fluorescence in the presence of MAβ40 (the 40-residue β-amyloid peptide with an additional N-terminal methionine) without affecting the lag time or elongation rate (Fig. 2*F*), consistent with the known ability of EGCG and ThT to compete for the same binding site (33).

### Enhancement of backbone and side-chain dynamics within fibrillar apoA-I

**Figure 3.**
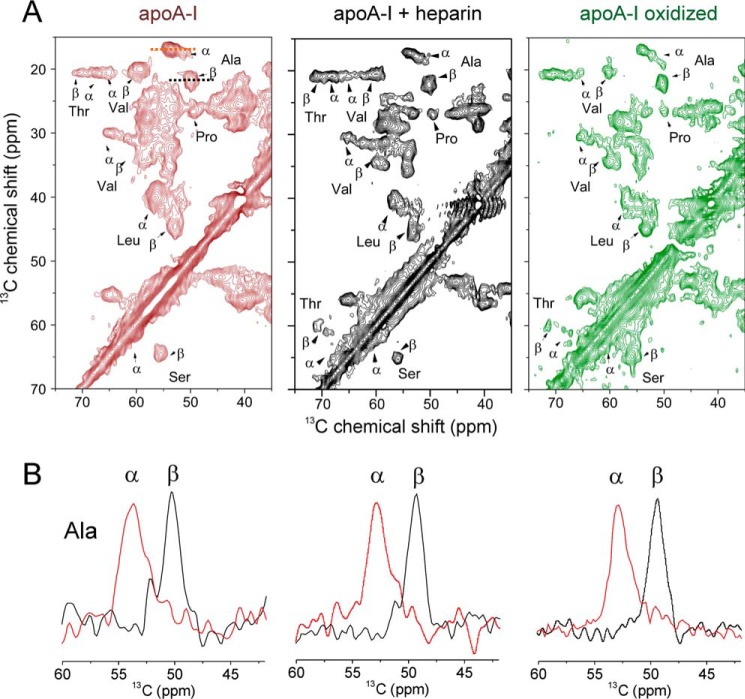
**ssNMR suggests a structural similarity of apoA-I fibrils formed under different conditions.** *A,* 2D ^13^C–^13^C solid-state NMR spectra (obtained with 20 ms of DARR mixing) of uniformly ^13^C-labeled apoA-I aggregates formed at pH 4 in the absence of heparin (*left panel*), in the presence of heparin (*center panel*), or at pH 6 after oxidation (*right panel*). Key assigned cross-peaks are labeled. *B,* horizontal 1D slices through each 2D spectrum at the frequencies of the alanine Cα–Cβ cross-peaks, as denoted by the *dashed lines* in *A. Red peaks* occur at the expected chemical shift of alanine within α-helices, and *black peaks* occur at the expected chemical shift of alanine within β-strands.

A more detailed analysis was next carried out to determine whether EGCG modulates the structure of the apoA-I fibrils as it does for other fibrillar proteins (28, 29, 33, 44). Solid-state NMR (ssNMR) has recently revealed interesting structural features of apoA-I fibrils in which the duplication of some cross-peaks in 2D ^13^C–^13^C spectra suggest that the fibrils comprise a mixture of α-helical elements and new amyloid-like β-sheet elements within the fibril architecture (12). 2D ^13^C–^13^C magic angle spinning (MAS) ssNMR spectra of ^13^C-labeled apoA-I fibrils (Fig. 3*A*), prepared at pH 4 alone or in the presence of heparin or at pH 6 after protein oxidation by hydrogen peroxide, exhibit similar features, including the same duplication of peaks for specific amino acids, including alanine, threonine, leucine, and valine. The duplicated peaks occur in approximately the same intensity ratios for each sample, as shown by the slices through the Cα–Cβ cross-peaks for alanine (Fig. 3*B*), suggesting that the α-helical and β-sheet elements, which give rise to the differences in chemical shift observed (12), occur within a single, common fibril architecture (see “Discussion”).

**Figure 4.**
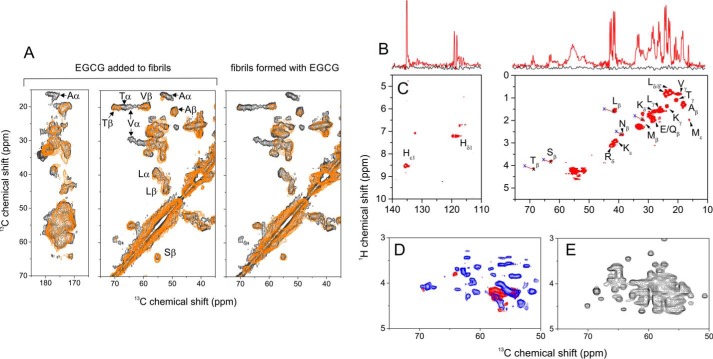
**Increased protein mobility after treatment of apoA-I fibrils with EGCG.** *A,* 2D ^13^C–^13^C solid-state NMR spectra (obtained with 20 ms of DARR mixing) of uniformly ^13^C-labeled apoA-I aggregates. The spectrum of fibrils prepared from 36 μm monomer (*black*) is shown overlaid with spectra of pre-formed fibrils after addition of 36 μm EGCG (*left panel, orange spectrum*) or overlaid with spectra of fibrils formed from 36 μm monomer in the presence of 36 μm EGCG (*right panel, orange spectrum*). *B,* regions of 1D ^13^C INEPT ssNMR spectra of insoluble aggregates (*black*) and aggregates after exposure to EGCG (*red*). *C,* corresponding regions of a 2D ^1^H–^13^C INEPT spectrum of EGCG-treated fibrils (prepared at pH 4 alone) labeled with assignments for specific amino acids. The *blue crosses*, linked by *red lines* to the resonances for Thrβ (T_β_), Serβ (S_β_), Leuβ (L_β_), Asnβ (N_β_), and Metβ (M_β_), represent the expected ^1^H and ^13^C chemical shifts for those amino acids in β-sheet regions. *D,* spectrum of fibrils formed alone from 36 μm apoA-I monomer and treated with equimolar EGCG (*red*) or fibrils formed in the presence of 72 μm heparin and treated with 36 μm EGCG (*blue*). *E,* simulated 2D ^1^H–^13^C INEPT spectrum (^1^Hα–^13^Cα region only) based on the predicted ^1^H and ^13^C chemical shifts of all 243 residues of apoA-I in an α-helical structure.

After adding EGCG to apoA-I fibrils formed at pH 4 in the absence of heparin, several cross-peaks disappear from the ^13^C–^13^C spectrum, specifically the cross-peaks assigned to valine, threonine, and alanine in α-helical environments and the proline Cγ–Cδ cross-peak (Fig. 4*A*, *left panel, red spectrum*). The remaining cross-peaks occur at the same positions as in the spectrum of untreated apoA-I fibrils (Fig. 4*A*, *left panel, black spectrum*). The changes in the spectrum can be interpreted as a selective structural remodeling of the fibrils, caused by exposure to EGCG. The same cross-peaks disappear from the spectrum of apoA-I fibrils assembled in the presence of EGCG (Fig. 4*A*, *right panel*), indicating that similar fibril remodeling occurs regardless of whether EGCG is present at the onset or added at the end point of aggregation.

An explanation for these observations is that EGCG increases protein mobility over selective regions within the fibrils; this is because cross-peaks in the ^13^C–^13^C spectrum arise from dipolar coupling between ^13^C nuclei and if the couplings are reduced by increased motional fluctuations then the cross-peaks can weaken in intensity or disappear. Increased mobility was tested using ^1^H–^13^C INEPT ssNMR, which detects signals only from dynamic regions of fibrillar proteins (45). A 1D INEPT spectrum of EGCG-treated fibrils reveals several sharp peaks attributable to the mobile regions of the protein (Fig. 4*B*, *red*), but the 1D INEPT spectrum of fibrils in the absence of EGCG fails to detect any signals after 24 h of measurement (Fig. 4*B*, *black*). These results indicate protein mobility within the untreated fibrils is enhanced after exposure to EGCG. A 2D extension of the ^1^H–^13^C INEPT experiment on the EGCG-treated fibrils enables some of the peaks to be assigned to specific amino acids (Fig. 4*C*). Most of the resonances occur in the amino acid side-chain region of the spectrum (principally from methyl-bearing or polar/charged side chains), and there is much less signal intensity in the Cα/Hα region, which implies that most of the protein backbone remains relatively constrained. The resolved ^13^Cβ chemical shifts of the mobile leucine, threonine, asparagine, alanine, and serine side chains are consistent with these residues occupying an α-helical structure (Fig. 4*C* and Table 2).

**Table 2:** **Measured chemical shifts from the ^1^H-^13^C INEPT SSNMR spectrum of apoA-I fibrils treated with EGCG and mean predicted chemical shifts for the given amino acids in α-helical and β-sheet environments** Chemical shifts are reported relative to tetramethylsilane.

Amino acid	^13^*C*β *chemical shift*	
	Measured	Predicted (α-helix/β-sheet)
	ppm	
Ala	18.23	18.30/21.72
Asp	39.72	40.50/42.78
Leu	41.21	41.40/44.02
Met	31.72	31.70/34.34
Ser	62.69	62.81/65.39
Thr	68.68	68.64/70.82

We next obtained a 2D ^1^H–^13^C INEPT spectrum of EGCG-treated apoA-I fibrils formed in the presence of heparin (Fig. 4*D*, *blue*). Surprisingly, many more signals are observed than are seen in the spectrum of EGCG-treated fibrils formed in the absence of heparin (Fig. 4*D*, *red*), particularly in the backbone Hα/Cα region shown in Fig. 4*D*. This observation is consistent with even greater mobilization of the fibrils formed when heparin is present, including enhanced dynamics of the protein backbone residues. Heparin and EGCG therefore appear to have an unexpected synergistic effect on increasing apoA-I mobility within protein fibrils. A simulated INEPT spectrum (Cα–Hα region only) based on all 243 apoA-I residues in an α-helical environment (Fig. 4*E*) implies that one would expect many more resonances to be observed if the protein was fully mobilized to an equal extent across the entire sequence.

### EGCG remodels heparin-promoted apoA-I fibrils into oligomers

**Figure 5.**
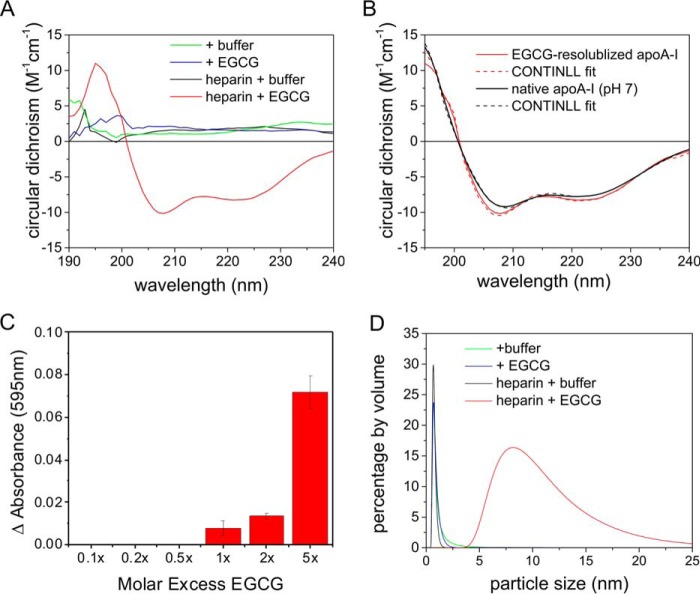
**Structure and size of EGCG-remodeled apoA-I fibrils.** *A,* far-UV CD spectra of the soluble apoA-I remaining in solution after addition of buffer or EGCG followed by removal of insoluble fibrils. The color scheme corresponds to fibrils formed in the absence of heparin and incubated with McIlvaine buffer (*green*) or with EGCG (*blue*) and fibrils formed in the presence of heparin incubated with buffer only (*black*) or with EGCG (*red*). *B*, comparison of the CD spectrum of EGCG-treated fibrils in the presence of heparin with the spectrum of soluble native apoA-I at pH 7 (*black*). The *dotted lines* indicate the best fits to the spectra obtained with the CONTINLL algorithm. *C,* analysis using the Bradford reagent indicates that the soluble protein released from fibrils formed in the presence of heparin increases with increasing EGCG concentration up to a 5-fold molar excess over apoA-I. *D,* DLS of the supernatant following centrifugation and removal of buffer-treated apoA-I aggregates and apoA-I aggregates treated with EGCG.

We next examined whether the increased mobility of apoA-I aggregates could originate from resolubilization of the fibrils by EGCG. CD spectroscopy was used to detect any structured soluble protein that may be released from the fibrils after binding EGCG. Fresh samples of fibrils formed (at pH 4) after 3 days were incubated with equimolar EGCG for 12 h, and the remaining insoluble material was removed by centrifugation before analysis of the supernatant by CD. For control experiments, the EGCG solution was replaced with McIlvaine buffer alone. Little signal was observed in the far-UV CD spectra of the control samples or in the spectra of EGCG-treated fibrils formed in the absence of heparin to which EGCG had been added (Fig. 5*A*), and reliable analysis of the secondary structure could not be performed. Hence, the concentration of soluble protein is low in these samples, and most of the protein was removed in the insoluble fraction. In marked contrast, when fibrils formed in the presence of heparin are treated with EGCG, the supernatant produces a strong signal in the far-UV CD that is characteristic of structured, soluble protein with α-helical content (Fig. 5*A*, *red solid line*). Quantitative analysis of the spectrum using the CONTINILL fitting algorithm (Fig. 5*B*, *red lines*) indicates that the structure of the soluble material is ∼75% α-helical. The CD spectrum of the native protein (Fig. 5*B*, *black lines*), which is stable in solution at pH 7 (12), indicates a similar helical content of 68%. Hence, EGCG results in the shedding of molecules with a predominantly α-helical structure from fibrils formed with heparin, but it is not clear whether the mobilized protein reverts to its native structural state. Dynamic light-scattering (DLS) analysis of the EGCG-treated apoA-I fibrils revealed species with a hydrodynamic radius distribution of 5–25 nm results from dissociation of fibrils assembled with heparin, although none of the other buffer- or EGCG-treated samples released soluble species of this size (Fig. 5*D*). No species larger than 25 nm were released from the fibrils. Together, the ssNMR, CD, and DLS data indicate that EGCG remodels insoluble apoA-I fibrils into soluble α-helical species much more readily when the fibrils have formed in the presence of heparin.

**Figure 6.**
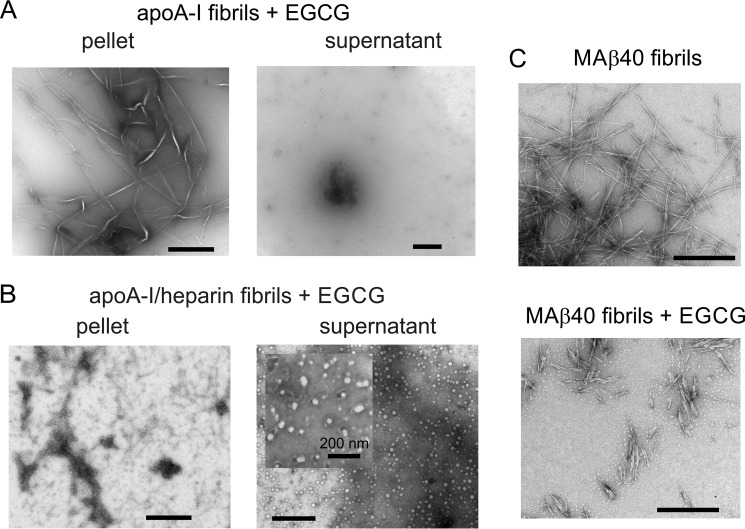
**Morphology of EGCG-remodeled apoA-I fibrils.** *A,* negative stain TEM images of EGCG the insoluble (*pellet*) and soluble (*supernatant*) fractions of apoA-I aggregates formed alone and then exposed to EGCG. *B*, TEM images of the insoluble and soluble fractions of apoA-I aggregates formed in the presence of heparin and then exposed to EGCG. *C,* TEM images of MAβ40 fibrils in the absence or presence of EGCG. In each case, fibrils were formed from 36 μm protein monomer alone or in the presence of 72 μm heparin, to which was added buffer or EGCG to a final concentration of 36 μm. All *scale bars* = 500 nm unless indicated otherwise.

The EGCG-treated fibrils (formed alone or with heparin) were next centrifuged, and the morphologies of the soluble species in the supernatant and the insoluble material in the pellet were visualized using negative stain TEM. For the fibrils formed alone, exposure to EGCG does not disrupt the fibrillar morphology within the insoluble fraction, and the soluble fraction is virtually free of visible protein aggregates in the 5–25-nm range (Fig. 6*A*). By contrast, fibrils formed in the presence of heparin are remodeled by EGCG into small, granular structures that remain insoluble, whereas a proliferation of soluble spherical oligomer-like species, 20–30 nm in diameter, is now also present (Fig. 6*B*). No such species of apoA-I were observed at any point in the absence of EGCG. Indeed, apoA-I assembly into fibrils is instantaneous in the presence of heparin at pH 4 and is unlikely to proceed via oligomeric intermediates. The combined effect of heparin and EGCG appears to extensively remodel the apoA-I aggregates into unique α-helical oligomers that are otherwise not observed in the aggregation pathway. In the absence of heparin, the effect of EGCG is less pronounced and more selective for specific regions of the protein. For comparison, we also visualized the effect of EGCG on Aβ40 fibrils (formed by seeding their growth in the absence of heparin). The results showed that fibrils are fragmented into smaller, spear-like structures after treatment with EGCG, as reported previously (29), although the smaller oligomeric and amorphous species observed in the same work are not observed here (Fig. 6*C*).

**Figure 7.**
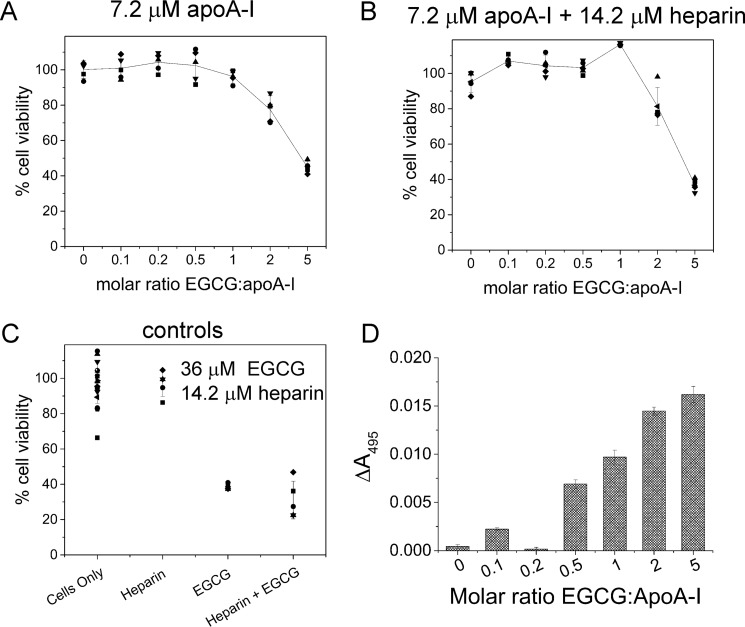
**Effect of EGCG-treated apoA-I fibrils on the viability of human umbilical artery endothelial cells.** *A,* effect on cell viability of apoA-I fibrils after exposure to different concentrations of EGCG. *B,* effect on cell viability of apoA-I fibrils formed in the presence of heparin after exposure to different concentrations of EGCG. *C,* control measurements of cell viability after the addition of heparin, EGCG, or a mixture of both. Means and standard errors are shown for *n* = 5 for each sample group except for the control cells only, for which *n* = 15. *D,* release of heparin from apoA-I fibrils into aqueous solution by the addition of different concentrations of EGCG. Fibrils were formed from 36 μm apoA-I in the presence of 72 μm heparin doped with 1% w/w fluorescein heparin conjugate, which was detected by absorbance at 495 nm.

We next investigated whether the soluble apoA-I aggregates released by EGCG are toxic to cells at varying molar ratios of EGCG to protein. A cell viability assay using the cellular dehydrogenase-sensitive dye WST-8 was used to assess the cytotoxicity of EGCG-treated apoA-I fibrils to human umbilical artery endothelial cells (Fig. 7, *A* and *B*). Insoluble fibrils (7.2 μm monomer equivalent), formed in the absence or presence of 14.4 μm heparin, were treated with EGCG up to a 5-fold molar excess (*i.e.* 36 μm) of the polyphenol over the protein, and the solutions were added to the cells after removal of the extant insoluble material. Cell viability is not impaired when apoA-I fibrils are exposed to equimolar or lower concentrations of EGCG, and this is apparent whether or not apoA-I fibrils are formed alone or with heparin (Fig. 7, *A* and *B*). Fibrils treated with higher concentrations of EGCG (2–5-fold excess) do show some impairment of cell viability (Fig. 7, *A* and *B*). However, control measurements on cells treated with 36 μm EGCG, 14.2 μm heparin, or 36 μm EGCG and 14.2 μm heparin together, all in the absence of apoA-I, indicate that the cytotoxic effect at higher EGCG concentrations is a direct result of excess polyphenol and is not related to the presence of apoA-I (Fig. 7*C*). ssNMR, DLS, CD, and TEM all agree that apoA-I fibrils formed in the presence of heparin release soluble oligomers after treatment with equimolar EGCG. Here, the data indicate clearly that the soluble oligomers are not cytotoxic.

### EGCG interacts with monomeric apoA-I but not with heparin

**Figure 8.**
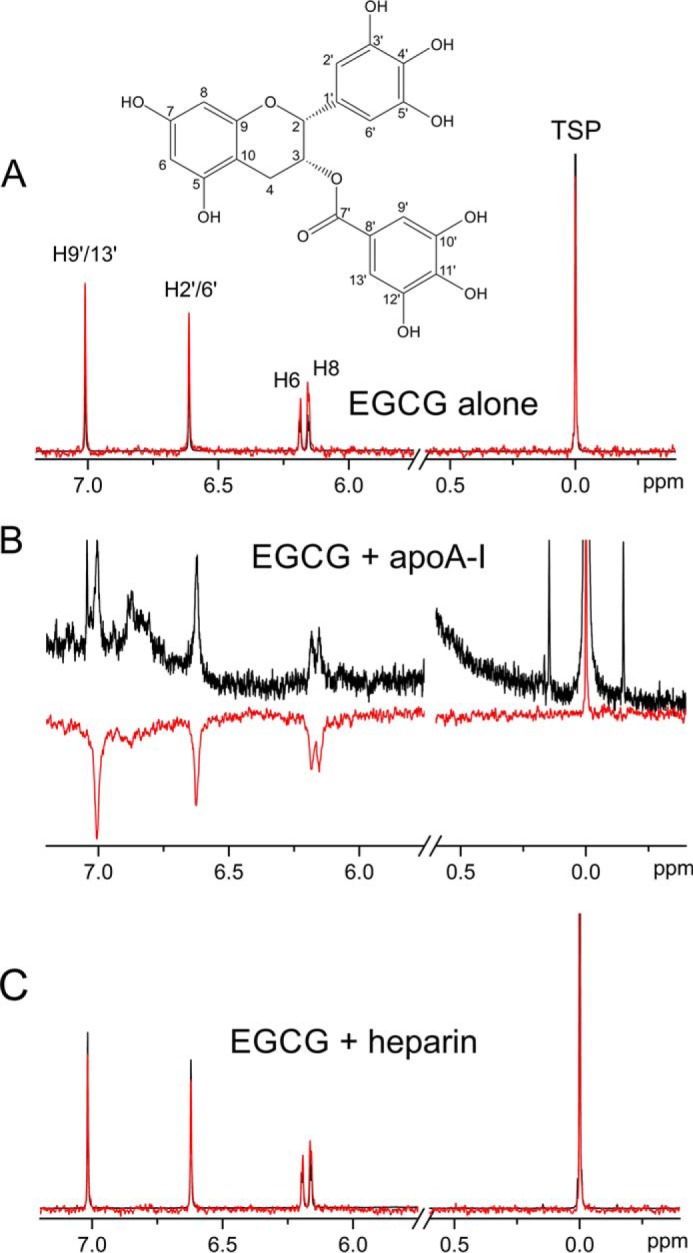
**NMR detection of EGCG interactions with apoA-I and heparin.** 1D ^1^H NMR spectrum (*black*) and water LOGSY spectrum (*red*) are shown for EGCG (720 μm) either alone (*A*), in the presence of apoA-I (36 μm) (*B*), or in the presence of heparin (36 μm) (*C*).

Exposure of apoA-I fibrils formed in the presence of heparin releases heparin into the aqueous phase in an EGCG concentration-dependent manner (Fig. 7*D*). We therefore investigated whether EGCG interacts with heparin as well as with the monomeric protein. A solution-state WaterLOGSY NMR experiment was used to detect EGCG binding to heparin and, separately, to monomeric apoA-I. In the WaterLOGSY experiments, bulk water magnetization is transferred to EGCG if it binds to the larger protein or polysaccharide molecule, resulting in resonances for EGCG that are of opposite sign to noninteracting compounds. We here used the internal reference trimethylsilylpropanoate (TSP) as the nonbinding molecule, comparing the sign of the peak at 0 ppm with EGCG aromatic resonances. WaterLOGSY spectra of EGCG alone and in the presence of heparin or apoA-I were compared with the corresponding 1D ^1^H spectra (Fig. 8). As expected, peaks from TSP and EGCG are narrow and have the same sign in the 1D and WaterLOGSY spectra in the absence of either of the macromolecules (Fig. 8*A*). In the presence of apoA-I, the peaks from EGCG broaden and take the opposite sign to the TSP peak in the WaterLOGSY spectrum (Fig. 8*B*), consistent with EGCG-protein binding, whereas the spectra of EGCG in the presence of heparin do not indicate an interaction between the two molecules (Fig. 8*C*).

**Figure 9.**
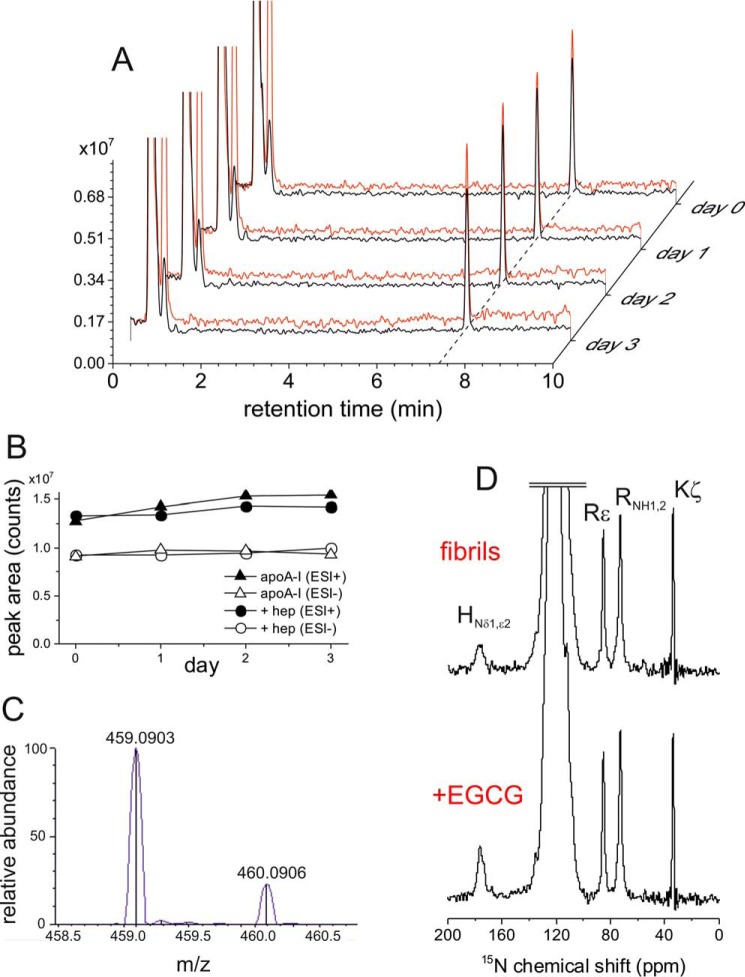
**EGCG interacts with apoA-I in the unmodified polyphenol form.** *A,* LC-MS chromatogram of a solution of EGCG incubated with apoA-I for 3 days (after removal of insoluble material), containing positive mode XIC 459.0922 *m*/*z* (*black*), and negative mode XIC 457.0776 *m*/*z* (*red*). *B,* peak areas measured from chromatograms (positive and negative mode XIC) after incubation of EGCG and apoA-I ± heparin for 0 and 1–3 days. *C,* example of electrospray ionization + high resolution MS mass assignment of EGCG at *t*_R_ = 7.65 min; 459.0903 *m*/*z* measured, 459.0922 *m*/*z* predicted for the C22H18O11 [M + H]^+^ ion. *D,* proton-decoupled ^15^N CP-MAS NMR spectra of uniformly ^15^N-labeled apoA-I fibrils (prepared at pH 4 in the absence of heparin) before (*top panel*) and after (*bottom panel*) incubation with EGCG for 24 h.

Finally, we investigated whether EGCG interacts with apoA-I as the unoxidized polyphenol or in the form of oxidized quinones. The reactive form of EGCG influences whether the interaction with apoA-I is covalent or noncovalent. EGCG is able to undergo auto-oxidation, generating quinones and superoxide, which in turn promotes further EGCG oxidation (46). The quinones can self-react to form polymeric species via intermolecular cross-linking and can also react covalently with available SH or NH_2_ groups of proteins (47). Under oxygenated conditions in solution at pH 7.4, EGCG was shown to be fully oxidized after a 6-h incubation period (33). Here, we assessed whether the low pH conditions used in the aggregation and re-solubilization studies protected against EGCG oxidation. EGCG polyphenol absorbs maximally at ∼275 nm, but oxidation above pH 7 results in a color change and shift in the absorbance maximum to 320 nm (48). Here, at pH 4 the maximum absorbance of EGCG alone in solution (72 μm) remained unchanged for 3 days with no evidence of additional absorbance bands appearing above 320 nm (data not shown), indicating that the polyphenol alone remains stable under these conditions. We next used LC-MS to determine whether EGCG undergoes oxidation over time in the presence of apoA-I. The LC-MS chromatograms for fresh and aged (up to 3 days) solutions of EGCG and apoA-I, with or without heparin showed an intense peak at 7.65 min (Fig. 9*A*). The peak area does not decrease significantly when measured after incubation of EGCG and apoA-I for 1–3 days (in fact, a slightly unexplained increase was observed), and no additional peaks consistent with oxidation or degradation products can be seen (Fig. 9*B*). The [M + H]^+^ and [M − H]^−^ peaks in the positive and negative polarity scans, respectively, were used to obtain extracted ion chromatograms (XIC) from which peak integration values were obtained. For both positive and negative ionization modes, the base peak observed at 7.65 min was assigned to a commonly observed adduction of unmodified EGCG at 459.0903 and 457.08 *m*/*z*, respectively (Fig. 9*C*). Finally, we checked whether fibrils of apoA-I could be covalently modified by EGCG, using ssNMR and ^15^N-labeled protein (in the absence of heparin). The ^15^N MAS NMR spectrum of apoA-I fibrils alone exhibit clear peaks from the lysine and arginine NH_2_ groups (Fig. 9*D*), which remain unchanged when the fibrils were incubated with EGCG for 1 day. A decrease in the lysine peak intensities and the appearance of peaks at higher chemical shifts (>120 ppm for Schiff bases) would be expected if the primary amines were covalently modified. We therefore conclude that EGCG does not oxidize or covalently modify apoA-I under the conditions applied here.

## Discussion

In the above experiments, we set out to investigate whether EGCG exerts a potentially protective effect on apoA-I amyloid that is associated with atherosclerosis, as reported for several other amyloidogenic proteins associated with disease (49, 50). Studies on Aβ40/42, α-synuclein, amylin, huntingtin, and transthyretin have all shown a common ability of EGCG to bind to the oligomeric and multimeric forms of amyloid, to inhibit fibril assembly, and to prevent formation of toxic structures (28, 29, 51–55). In the case of both Aβ40/42 and α-synuclein, EGCG prevents fibrillogenesis by binding preferentially to unfolded protein, directing assembly toward off-pathway nontoxic oligomers, and thereby preventing formation of toxic oligomers and protofibrils (29). When added to pre-formed aggregates, EGCG can also alter their β-sheet conformation and convert aggregates into small, nontoxic amorphous aggregates (28, 29). Remodeling of amyloid from several different proteins may also involve auto-oxidation of EGCG, which can result in covalent modification of free amine groups, although this mechanism is not the major driving force for the structural remodeling (33). Here, we have identified similarities but also reveal a remarkable and hitherto unknown synergy between the effect of this polyphenol and the GAG heparin on the behavior of an amyloid aggregate.

To rationalize our observations, it is convenient to consider the structural features of apoA-I aggregates at the molecular level. ApoA-I forms ThT-responsive aggregates under oxidative or acidic conditions, and the process is accelerated by the GAG analogue heparin, which co-localizes with the fibrils (Fig. 1). In the latter regard, apoA-I behaves similarly to Aβ, α-synuclein, and amylin, all of which assemble into amyloid more rapidly in the presence of heparin (42, 56–59). In some cases, heparin decorates the surface of the fibrils, such as in salmon calcitonin fibrils (60) and in the 3Q fibrils of Aβ40 (42, 45, 59), whereas in other cases heparin does not form a stable complex with the insoluble fibrils (61). The apoA-I aggregates formed under the conditions employed here are predominantly fibrillar (∼10 nm diameter), which form alongside amorphous material that is known to form around the native protein's pI of 5.2 (62). The rate of fibril formation is rapid at pH 4, which is a much more acidic pH than that measured in *ex vivo* lesions (14). However, the fibrillar end point appears to be structurally identical to fibrils formed at a higher, more physiological pH after myeloperoxidase-catalyzed oxidation or after incubation of unmodified protein for longer periods (12). The low pH conditions applied here are for the purpose of expediting the fibrillar end point rather than for replicating the lesional microenvironment. ssNMR spectra reveal the co-existence of α-helical and β-sheet secondary structural elements in approximately the same proportions in the apoA-I aggregates, regardless of the aggregation conditions employed (Fig. 4). We believe that both structural elements occur within a single fibrillar architecture, such that each protein molecule within an aggregate possesses both β-sheet and α-helical regions. An alternative explanation that apoA-I assembles via two divergent pathways into discrete assemblies of extensively β-sheet protein and predominantly α-helical protein is also possible.

EGCG is the major polyphenol component of green tea known bind to pre-formed aggregates of apoA-I. EGCG has been shown to interact with Ile, Phe, and Tyr residues, preventing the hydrophobic contacts required for conversion to a stable β-sheet conformation and enabling disaggregation of pre-formed amyloid fibrils (63). Aβ40 assembles into fibrils via conformationally labile states, potentially enabling access of EGCG to block hydrophobic contacts, but the compact native structure of lipid-free apoA-I (64) and its ability to form abundance of hydrophobic contacts stabilizing the native helical bundle may overcome any inhibitory effect that EGCG may exert.

The most striking outcome of this work is the effect of heparin on the ability of EGCG to restructure aggregates of apoA-I. In the absence of heparin, EGCG selectively mobilizes the alanine, valine, and threonine side chain within α-helical regions, and proline residues are also affected (Fig. 4). Proline residues occur throughout the apoA-I sequence and punctuate the helical regions with hinges, enabling the protein to form the annular belt-like structure associated with HDL (65). The INEPT ssNMR experiments indicate that the amino acid side chains are mobilized to a greater extent than the backbone (Fig. 4*C*), which implies that EGCG acts largely peripherally, leaving the stable β-sheet core of the fibrils intact. When EGCG is added to fibrils assembled in the presence of heparin, its remodeling effect is amplified significantly, yielding an abundance of spherical oligomers ∼20 nm in diameter that are nontoxic to human endothelial cells.

How do heparin and EGCG cooperate to remodel apoA-I fibrils? A direct interaction between EGCG and heparin was ruled out by WaterLOGSY NMR experiments. Heparin was also shown to enhance the aggregation kinetics of apoA-I (Fig. 2*D*), likely by the high local ionic strength micro-environment of heparin combined with the directionality and periodicity of its anionic groups that may act as a template for apoA-I assembly (66). The ssNMR spectra do not support major structural differences in the fibrils formed with or without heparin (Fig. 3), but it is possible that subtle structural modifications by heparin could open up additional binding sites for EGCG. Alternatively, the partial mobilization of the fibrils by EGCG seen in the absence of heparin could be amplified by heparin when bound to the fibril surface.

The lack of cellular toxicity of the soluble oligomers promoted by EGCG in the presence of heparin, when taken together with their absence during the fibril assembly pathway, suggests that these are unique species formed specifically by the action of EGCG on the fibrils, rather than a regeneration of on-pathway, toxic annular oligomers that are observed for other amyloidogenic peptides and proteins (67). Annular species have been observed when methionine-oxidized apoA-I undergoes aggregation at pH 6 (68), but these are not formed reproducibly and are in much smaller amounts than the profusion of oligomers observed here. Why the oligomers are not toxic is unclear. EGCG binds to pre-formed oligomers of α-synuclein and protects against their inherent cytotoxicity by inhibiting oligomer–membrane interactions (69). A similar mechanism might underlie the observations on apoA-I but cannot easily be tested because of the inability to prepare pre-formed oligomers in the absence of EGCG.

The ability of EGCG to reproduce the observed effects *in vivo* depends on the available concentration of EGCG in blood and in the aortic intima. EGCG reaches maximum plasma concentration 2 h after drinking a cup of green tea, but the steady-state concentration may be much higher for frequent green tea drinkers (70). Studies with human serum albumin (HSA) indicate that EGCG binds to two high-affinity sites (*K_D_* = 22 μm) and several additional low-affinity surface sites (mm *K_D_*), suggesting that plasma concentrations of HSA (∼600 μm) can bind up to 98% of EGCG (71). This would account for the slow metabolism of EGCG but could also restrict its availability for interactions with amyloid proteins, including Aβ, α-synuclein, and apoA-I. However, as Eaton and Williamson point out in their recent work (71), the weaker (millimolar) binding implies fast off-rates and the weakly bound EGCG would dissociate at a rate of 100 s^−1^ or faster (assuming the association rate is at the diffusion-limit). Hence, the exchange of EGCG between HSA and tissue is likely to be rapid. With regard to the atherosclerotic environment, HSA and its cargo can enter the proteoglycan-rich intimal layer of the aorta from the luminal and adventitial sides (72), and the concentration of HSA in the interstitial space is approximately half that in plasma (73). Further detailed experiments, beyond the scope of this work, will be required to establish whether the local acidic environment of atheromatous tissue enhances the release of sufficient EGCG from HSA for interaction with fibrillar apoA-I. It is interesting to note, however, that EGCG affinity for BSA decreases in acidic solution (74).

In summary, we demonstrate here an unusual interplay between apoA-I, EGCG, and heparin that, together, mobilize fibrils and result in the shedding of nontoxic oligomers that does not occur in the presence of either ligand alone. The results add a layer of complexity in considering the effects of small molecules on the progress of amyloid assembly and amyloid-associated cytotoxicity in a cellular context, wherein other components such as GAGs may affect the outcome of administration of a small molecule. Indeed, a number of natural GAG variants have been identified as co-localizing with amyloid plaques, including chondroitin sulfate (75–77), dermatan sulfate (78), and keratan sulfate (79), all of which, like heparin and HS, contain polysulfated disaccharide units. Whether fibril mobilization is a benefit or a threat may also be reconsidered, where reduction of fibril load may be of greater benefit than the production of small amounts of oligomers, which may be degraded, further dissociated by binding to molecular chaperones *in vivo*. Whatever the outcome, the results presented suggest that it may now be appropriate to re-evaluate the wider, potentially protective effects of EGCG as an amyloid-remodeling agent, by considering the synergistic effects of GAGs and other ubiquitous co-factors of amyloid *in vivo*.

## Materials and methods

### Protein expression and fibril formation

Expression of N-terminally His-tagged apoA-I was carried out by following previously published methods (80, 81). A pNFXex expression vector coding for human apoA-I with an N-terminal His tag (kindly provided by Dr. M. Oda, Oakland Research Institute) was transformed into *Escherichia coli* BL21 (DE3) cells (Agilent Technologies) and grown at 37 °C in LB media containing 100 μg/ml ampicillin (Melford Laboratories). The plasmid construct expresses apoA-I with an E2D mutation enabling removal of the His tag by cleavage of the acid-labile Asp-2–Pro-3 peptide bond with formic acid, leaving the native residues 3–243. The remaining expression and purification methods are described in Ref. 12.

ApoA-I fibrils were formed from up to 36 μm apoA-I incubated in McIlvaine buffer (165 mm Na_2_HPO_4_, 17.6 mm citrate, pH 4), typically for 3 days (unless specified otherwise), alone or in the presence of a 2-fold molar excess of heparin (IdoU(2S)-GlcNS(6S) 14–15 kDa, >70%, Iduron). Expression of MAβ40 and fibril formation by seeding with the 3-fold symmetrical (3Q) morphology was carried out as described previously (45).

### Isolation of polyphenol compounds from green tea

Green tea (2 g, Twining's^TM^ pure) was added to 40 ml of water. The solution was microwaved at 900 watts of power for two cycles of 30 s, followed by four cycles of 15 s with a minute between each heating. The solution was filtered through a 1-μm filter paper (Whatman) and filtered with a 20-μm Corning syringe filter. The solution was then freeze-dried until further use.

HPLC analysis of green tea compounds is as follows: a stock solution of the green tea extract was prepared by dissolving the lyophilized material in water (7.2 mg/ml) for analysis by reverse-phase HPLC. The pH of the solution was 7.5. A standard mixture of eight green tea catechins (each at 100 μg/ml) (Sigma, UK) was used as a reference set to assist in the assignment of HPLC peaks. Separation was performed on a NexeraX2 UHPLC (Shimadzu) system with a mobile phase of 0.1% orthophosphate in ultrapure water (A) or in acetonitrile (B), whereas the static phase consisted of a Shim-pack XR-ODS 2.2 μm, 3.0 × 50 mm) reverse-phase column. The green tea stock solution was injected after a further 10-fold dilution in water, and the standard catechin solution was injected without further treatment. The injection volume was 10 μl in both cases. The gradient elution, at a flow rate of 1 ml/min, consisted of 0–3 min (5% of B), 3–10 min (5–20% B), 10–13 min (20–50%), 13–13.1 min (50 to 5% B), and 13.1–20 min (5% B). Absorbance intensity was measured at 275 nm with a bandwidth of 4 nm.

For determination of green tea polyphenol binding to apoA-I, a 200-μl suspension of apoA-I fibrils (36 μm monomer equivalent) was centrifuged for 10 min at 12,000 × *g*. The green tea stock solution was diluted 200-fold in water, and 200 μl was added to the fibrillar pellets followed by incubation with agitation at 37 °C for 24 h. The fibrils were pelleted through centrifugation, and the supernatant was removed without further treatment for analysis by HPLC as described above.

### LC-MS analysis of EGCG

ApoA-I (36 μm) plus 72 μm EGCG was acidified at pH 4 in the presence and absence of 72 μm heparin, and fibrils were allowed to form over 3 days at 37 °C with shaking. At intervals of 0 and 1–3 days, the samples were centrifuged, and 50-μl aliquots of supernatant were taken for further testing by LC-MS. Mass spectra were acquired using a Shimadzu LC-MS-IT-TOF mass spectrometer. LC separations were performed using a Shimadzu NexeraX2 UHPLC instrument consisting of a DGU-20A5R degassing unit, two LC-30AD LC pumps, a SIL-30AC autosampler, and a CTO-20AC column oven. Separation was performed using a Shim-pack XR-ODS (3.0 × 50 mm, 2.2 mm) column with an oven temperature of 35 °C. The mobile phase was composed of water with 0.1% formic acid (A) and acetonitrile with 0.1% formic acid (B). A binary gradient elution of 0.0–3.0 min 5% B, 10.0 min 20% B, 10.1 min 50% B, 13.0 min 50% B, 13.1 min 5% B, 20.0 min 5% B was at a flow rate of 0.5 ml/min. High resolution mass spectrometric data were measured using an electrospray ionization probe with a curved desolvation line and a heat-block temperature of 200 °C, and an N_2_-nebulizing gas flow rate of 1.5 liters/min. Data acquisition was performed in both positive and negative ionization with polarity switching. A positive acquisition range of 100–1000 *m*/*z* with ion accumulation at 5.0 ms and a negative acquisition range of 200–1000 *m*/*z* with ion accumulation at 2.0 ms were shown. Samples were held in the autosampler at 5 °C while queued for analysis. The LC-MS/ion trap-TOF mass accuracy was calibrated with sodium trifluoroacetate clusters prior to analysis of the batch of samples. LC-MS data were analyzed using Shimadzu LC-MS solution software, and peak areas were calculated for the predicted *m*/*z* value of [M + H]^+^ ion and [M − H]^−^ ions in positive and negative ionization scan modes, respectively, using the Qualitative Peak Integration function in the LC-MS Postrun Analysis software.

### Binding of heparin to apoA-I fibrils

*In vitro* binding assays of apoA-I fibril-heparin binding were performed using an adaptation of a procedure described previously (59), in which the amount of GAG remaining unbound at different fibril/GAG concentration ratios was determined using *Bacteroides* heparinase I (New England Biolabs Ltd., UK). The heparinase enzyme cleaves heparin yielding oligosaccharide products containing unsaturated uronic acids, which can be detected using UV spectroscopy at 232 nm. Fibrils were prepared by acidification of apoA-I in the presence of heparin (Sigma), at the following protein/heparin molar ratios: 1:0.5, 1:1, and 1:2. In addition, samples of protein only and heparin only were prepared as controls. In all cases, the heparin concentration was kept at 1 mg/ml (72 μm). ApoA-I was initially solubilized in pH 7 McIlvaine's buffer together with heparin before carrying out a shift to pH 4 with the addition of concentrated HCl. Samples were incubated at 37 °C for 3 days with shaking to allow fibril formation prior to sedimentation of the protein. The supernatant was removed and returned to pH 7 with 5 m NaOH before assay. Samples were monitored at 30 °C, with *A*_232_ measurements taken every 10 s until maximal absorbance was reached. The amount of heparin remaining in solution was then calculated from a heparin calibration curve.

### Effect of green tea compounds on the aggregation of apoA-I

ThT fluorescence experiments were carried out as described in Ref. 12. Briefly, apoA-I (7.2 μm) was incubated with ThT alone and in the presence of 14.4 μm heparin. Samples (in triplicate) were incubated with no further additives, with pure green tea extract solution (6.6 ng/ml, roughly equivalent to a 2-fold molar excess of EGCG), or 14.4 μm EGCG. After measuring the fluorescence for 10 min, concentrated HCl was added to reduce the sample to pH 4 and induce aggregation. ThT fluorescence measurements were taken over 300 min at 1-min intervals.

### Binding of EGCG to apoA-I fibrils

The apoA-I (36 μm) was incubated alone or with increasing concentrations of EGCG at pH 4, 37 °C, for 3 days with agitation. After this time, the fibrils of apoA-I formed in the absence of EGCG were incubated for a further 24 h in the presence of EGCG. The insoluble material was removed by centrifugation at 13,400 rpm in a bench-top centrifuge. The absorbance of the supernatant at 274 nm (the wavelength of maximum absorption of EGCG) was measured and used to determine how much EGCG remained in solution by comparison with a standard curve of *A*_274_ for EGCG at a range of concentrations from 0.01 to 1 mm.

### Transmission EM

ApoA-I (36 μm), in the absence and presence of heparin (72 μm), was incubated at pH 4 at 37 °C with agitation for 3 days. Samples were centrifuged, and the pellets were washed several times with distilled water to remove buffer salts. Some pellets were resuspended in 36 μm EGCG and incubated overnight at 25 °C before removal of the bulk solution by centrifugation. Pellets were then diluted to 18 μm, before 10 μl was loaded onto carbon-coated copper grids and incubated for 30 s. Excess sample was removed by blotting. Grids (Agar Scientific Ltd., Stansted, UK) were washed two times with 10 μl of water. The grid was then stained by inverting it onto a 10-μl droplet of 2% (w/v) uranyl acetate and blotting, followed by addition of another 10-μl uranyl acetate droplet. After 30 s of staining, the grid was blotted and left to dry for 5 min at room temperature. The sample was then visualized on a JEOL JEM-1400 electron microscope at 100 kV. TEM images were calibrated in ImageJ (National Institutes of Health) using the TEM scale bar on each image. Calibrated lines were drawn to measure fibril width for a total of 25 instances per image. These instances could include up to three measurements of the same fibril at different positions in the image. A total of five images for apoA alone and five images of apoA + heparin was analyzed, resulting in an average fibril width of 10.7 ± 0.4 nm for apoA alone and 15.4 ± 1.3 nm for apoA + heparin. All measurements were then binned into 1-nm categories for data presentation.

### CD spectroscopy

ApoA-I (36 μm) alone or in the presence of heparin (72 μm) was incubated at pH 4 at 37 °C for 3 days with agitation. The samples were centrifuged, and the pellets washed several times with distilled water to remove buffer salts. The aggregated apoA-I was resuspended in McIlvaine buffer or McIlvaine buffer containing 36 μm EGCG. The samples were then incubated at 37 °C with agitation for 24 h and then centrifuged at 14,000 rpm to remove the insoluble aggregated protein. Spectra of the supernatants were acquired on a Chirascan Plus CD spectrometer between 180 and 260 nm with a bandwidth of 1 nm, using a path length of 0.2 mm. Background signals (*i.e.* from buffer, heparin, EGCG, or EGCG and heparin) were subtracted, and the spectra were analyzed using CDApps software (82). Fitting was performed using the CONTINLL algorithm.

### Dynamic light-scattering

ApoA-I (36 μm) in the absence and presence of heparin (72 μm) was incubated at pH 4 at 37 °C with agitation for 3 days. Insoluble fibrils were centrifuged and washed three times with distilled water to remove buffer salts. The fibrils were resuspended in McIlvaine buffer or McIlvaine buffer with 36 μm EGCG and incubated at 37 °C for 24 h. The insoluble aggregated material was removed through centrifugation at 14,000 rpm, and the supernatant was loaded into low-volume plastic cuvettes. DLS spectra were acquired on a Zetasizer Nano Zs instrument. The size distribution (in nanometers) by percentage volume was recorded and averaged over three scans.

### Solid-state NMR

Uniformly ^13^C-labeled apoA-I (5 mg at 36 μm) was incubated with agitation at pH 4 at 37 °C for 3 days alone or in the presence of 72 m heparin or with 72 μm heparin and 36 μm EGCG. Following the production of insoluble material, aggregates were harvested by centrifugation at 12,000 × *g* for 10 min. The aggregates were centrifuged into a zirconium 3.2 mm rotor with a Kel-F cap (Bruker, UK). In some samples, EGCG (36 μm) was added in a volume of 5 ml to the apoA-I samples and left for 24 h before transferring the pellet to the NMR rotor.

Two-dimensional ^13^C–^13^C ssNMR spectra were recorded at a magnetic field of 16.3 tesla on a Bruker Avance III 700 spectrometer, with a 3.2-mm HXY probe operating in double-resonance mode. Spectra were obtained with magic angle spinning at 14 kHz. Hartmann-Hahn cross-polarization was achieved with a 2-ms contact time, and 100 kHz proton decoupling with SPINAL-64 applied during signal acquisition. During the mixing time of 20 ms, the ^1^H nutation frequency was lowered to 14 kHz to achieve dipolar-assisted rotational resonance (DARR) mixing. A total of 256 *t*_1_ increments were recorded using the States-TPPI method for phase sensitivity, with 256 transients per increment. One- and two-dimensional refocused INEPT ssNMR spectra (83) and proton-decoupled ^15^N cross-polarization–magic angle spinning (CP-MAS) spectra were recorded at a magnetic field of 9.3 tesla on a Bruker Avance III 400 spectrometer. Spectra were obtained with magic angle spinning at 8 kHz. For CP-MAS, a contact time of 2 ms and proton spin-lock frequency of 63 kHz were applied, with proton decoupling at 83 kHz.

### Solution-state NMR

Proton spectra were recorded at a magnetic field of 9.3 tesla. Spectra were obtained for EGCG (360 μm) in 10 mm phosphate buffer, pH 7, alone or in the presence of heparin (36 μm) or apoA-I (36 μm). 1D gradient NOESY experiments were acquired with relaxation delays of 2 s, a mixing time of 10 ms, a presaturation power of 25 Hz, and 128 scans. Gradient strengths were the default for this sequence: GPZ1 50%, GPZ2 −10%. Spectral width was 8012.82 Hz, the offset on-resonance with the water peak, and 64k points (acquisition time 4.1 s). Spectra were zero-filled once and processed with a 0.3 Hz exponential line-broadening. WaterLOGSY spectra were acquired with relaxation delays of 1 s, a mixing time of 1.7 s, 2 dummy scans, and 128 scans. No spin-lock was applied to observe the receptor signals The selective inversion pulse for the NOESY block was a 7.5-ms Gaussian pulse, although that in the excitation sculpting block was a 2-ms sinc pulse (again, the prosol defaults). The spectral width was 6393.862 Hz, the offset on-resonance with the water peak, and 16k points (acquisition time 1.28 s). Spectra were zero-filled once and processed with a 1 Hz exponential line-broadening.

### Cell viability

Human umbilical artery endothelial cells (Sigma) were plated on 96-well plates at a cell density of ∼5000 cells per well and grown overnight at 37 °C to allow the cells to adhere. ApoA-I aggregates (72 μm) were formed over 3 days at pH 4 in the presence or absence of 144 μm heparin. Fibrils were pelleted and washed three times in pH 4 McIlvaine's to remove any unaggregated material before resuspending at the same concentrations. Samples were divided into equal volume aliquots, and EGCG was added at a range of concentrations to give molar ratios of 0, 0.1, 0.2, 0.5, 1, 2, and 5:1 EGCG/apoA-I, and left for a further 24 h, before the addition of 10-μl samples to the cells (100 μl assay volume). Following incubation at 37 °C for 48 h, 10 μl of the Cell Counting Kit (CCK-8) (Sigma) solution was added and incubated for a further 3 h prior to absorbance measurement at 450 nm.

## Author contributions

D. T., E. H., K. L. S., and S. E. R. investigation; D. T., G. A., D. R., and D. A. M. methodology; E. H. formal analysis; S. E. R. writing-original draft; D. A. M. conceptualization; D. A. M. supervision.

## Footnotes

5 The abbreviations used are:

Aβ: amyloid-β
Aβ40: amyloid β(1–40)
apoA-I: apolipoprotein A-I
DARR: dipole-assisted rotational resonance
DLS: dynamic light-scattering
ECG: epicathin-3-gallate
EGCG: epigallocathin-3-gallate
GAG: glycosaminoglycan
HDL: high-density lipoprotein
INEPT: insensitive nuclei enhanced by polarization transfer
MAS: magic angle spinning
CP-MAS: cross-polarization–magic angle spinning
TEM: transmission electron microscopy
ThT: thioflavin-T
TSP: trimethylsilylpropanoate
XIC: extracted ion chromatogram
ssNMR: solid-state NMR
HSA: human serum albumin.
